# miR-27a inhibits molecular adhesion between monocytes and human umbilical vein endothelial cells; systemic approach

**DOI:** 10.1186/s13104-022-05920-9

**Published:** 2022-02-10

**Authors:** Farhad Shaikhnia, Ghasem Ghasempour, Asghar Mohammadi, Mohammad Shabani, Mohammad Najafi

**Affiliations:** 1 Clinical Biochemistry Department, Faculty of Medical Sciences, Iran University of Medical Sciences, Tehran, Iran; 2 Cellular and Molecular Research Center, Iran University of Medical Sciences, Tehran, Iran; 3 Microbial Biotechnology Research Center, Iran University of Medical Sciences, Tehran, Iran; 4 Faculty of Medical Sciences, Tarbiat Modares University, Tehran, Iran

**Keywords:** SELP, JAM-B (2), SELE, miR-194, miR-27a, Adhesion, HUVEC cells

## Abstract

**Objective:**

The endothelial cells overexpress the adhesion molecules in the leukocyte diapedesis pathway, developing vessel subendothelial molecular events. In this study, miR-194 and miR-27a were predicted and investigated on the expression of adhesion molecules in HUVEC cells. The SELE, SELP, and JAM-B adhesion molecules involved in the leukocyte tethering were predicted on the GO-enriched gene network. Following transfection of PEI-miRNA particles into HUVEC cells, the SELE, SELP, and JAM-B gene expression levels were evaluated by real-time qPCR. Furthermore, the monocyte-endothelial adhesion was performed using adhesion assay kit.

**Results:**

In agreement with the prediction results, the cellular data showed that miR-27a and miR-194 decrease significantly the SELP and JAM-B expression levels in HUVECs (P < 0.05). Moreover, both the miRNAs suppressed the monocyte adhesion to endothelial cells. Since the miR-27a inhibited significantly the monocyte-endothelial adhesion (P = 0.0001) through the suppression of SELP and JAM-B thus it might relate to the leukocyte diapedesis pathway.

## Introduction

Atherosclerosis is the leading cause of coronary artery diseases (CAD), peripheral artery diseases (PAD), and cerebrovascular diseases (CVD) including myocardial infarction, stroke, and heart failure [1, 2]. Many studies showed that inflammatory events such as hypercholesterolemia, dyslipidemia, and hypertension are known as important risk factors of atherosclerosis [3, 4]. Moreover, the process of atherosclerosis progresses due to endothelial cell activation, monocyte recruitment, and macrophage polarization in the vessel sub-endothelial space [5, 6]. LDL-cholesterol, cytokines and cell damages play the key roles at the beginning of atherosclerosis [5]. The LDL particles accumulate in the intima of arteries and convert to oxidized-LDL (Ox-LDL) mainly due to Reactive Oxygen/Nitrogen Species (ROS/RNS) produced in various cells such as vascular smooth muscle cells (VSMCs), endothelial cells, and macrophages [4]. Ox-LDL and other inflammatory cytokines are able to activate endothelial cells and to induce the expression of adhesion molecules such as ICAM-1, VCAM-1, E-selectin (SELE), and P-selectin (SELP) on the cellular surfaces [7–10]. Inclacumab, a human monoclonal antibody against P-selectin inhibits the leukocyte rolling [11]. Furthermore, the monocyte diapedesis is a process mediated by junctional adhesion molecules (JAMs) [12–16]. JAMs belong to an immunoglobulin superfamily (JAM-A, B, and C) and express on both leukocyte and endothelial cells [17–19]. Following the monocyte diapedesis, some factors such as monocyte chemotactic protein-1 (MCP-1), macrophage colony-stimulating factor (M-CSF), and IL-8 affect macrophage polarization [4]. Macrophages scavenge the ox-LDL and convert to foam cells resulting in more recruitment of monocytes, lymphocytes, and SMCs into atheroma plaque [4]. Finally, macrophages by production of matrix metalloproteinase enzymes (MMPs) degrade the extracellular matrix (ECM), causing plaque remodeling [1–3, 5, 6]. In the other hand, microRNAs (miRNA/miR) are small nucleotides that affect the cellular pathways via the regulation of gene expression levels [13, 20, 21]. Many studies reported that microRNAs might retard atherosclerosis process by targeting some genes such as adhesion molecules [20, 22]. In this study, miR-194 and miR-27a were predicted using miRNA-gene databases and monocyte-endothelial adhesion was evaluated through the changes of P-selectin, E-selectin, and JAM-B gene expression levels.

## Main text

### Methods

#### Cell culture

Human umbilical vein endothelial cells (HUVEC; C554) were purchased from Pasteur Institute (Tehran) and were seeded and cultured in DMEM-F12 (containing FBS 10% (Gibco, Thermo Fisher Scientific-US) and Pen-strep 1% (Sigma-Aldrich Co., St Louis, MO, USA)) in normal conditions (5% CO2, 37 °C).

#### microRNA transfection

To deliver microRNAs into HUVEC cells, Polyethylenimine (PEI, Sigma Aldrich) was used. N/P ratio equal to 20 from each PEI-miR complex (containing miR-194, miR-27a, and scramble) was made and added to the culture medium of HUVEC cells for 4 h. Then, the cells were washed by PBS and were maintained for 24 h.

#### Flow cytometry

To evaluate miRNA transfection, the flow cytometry technique was used. After incubating the cells with PEI containing FITC-conjugated miRNA for 4 h, they were harvested and examined by flow cytometry technique (CyFlow, Partec, Germany).

#### RNA extraction, cDNA synthesis, and RT-qPCR

Total RNA was extracted by GeneAll-Hybrid-R RNA purification kit (Seoul, South Korea). cDNA was synthesized using Reverse Transcriptase HyperScript™ First-strand Synthesis Kit (Seoul, South Korea). Real Time-qPCR was accomplished by TB Green Premix Ex Taq (Tli RNase H Plus) (Takara, Seoul, South Korea) using StepOne instrument (Applied Biosystems, Foster City, CA, USA). The gene expression data were normalized with beta-actin (ACTB), a house-keeping gene, to access relative gene expression. The primers of SELE (F-AGAATCAGAAACAGGTGC and R-GATGGGTGTTGCGGTTTCAG), SELP (F-ACGCTGCATTTGACCCGAG and R- CCCAAACTCAGGAAACAGGGT), JAM-B (F-GACAAGAAGTGATGCGGGGA and R- ATGATGGAACTGCTGGAGCC), and ACTIN-B (F-GCAAGCAGGAGTATGACGA and R- CAAACAAATAAAGCCATGCCAATC) were designed and checked by primer blast server.

#### Monocyte-endothelial adhesion

Monocytes from healthy subjects were isolated from whole blood by RosetteSep™ Human Monocyte Enrichment Cocktail kit (STEMCELL Technologies Canada Inc.). After incubating (24 h), the miRNA-transfected HUVEC cells were cocultured with isolated monocytes. Then, the molecular adhesion was assessed by CytoSelect™ Leukocyte-endothelium Adhesion Assay kit (Cell Bio labs, Inc. San Diego, CA 92126 USA).

#### Gene and miRNA predictions

**Fig. 1:**
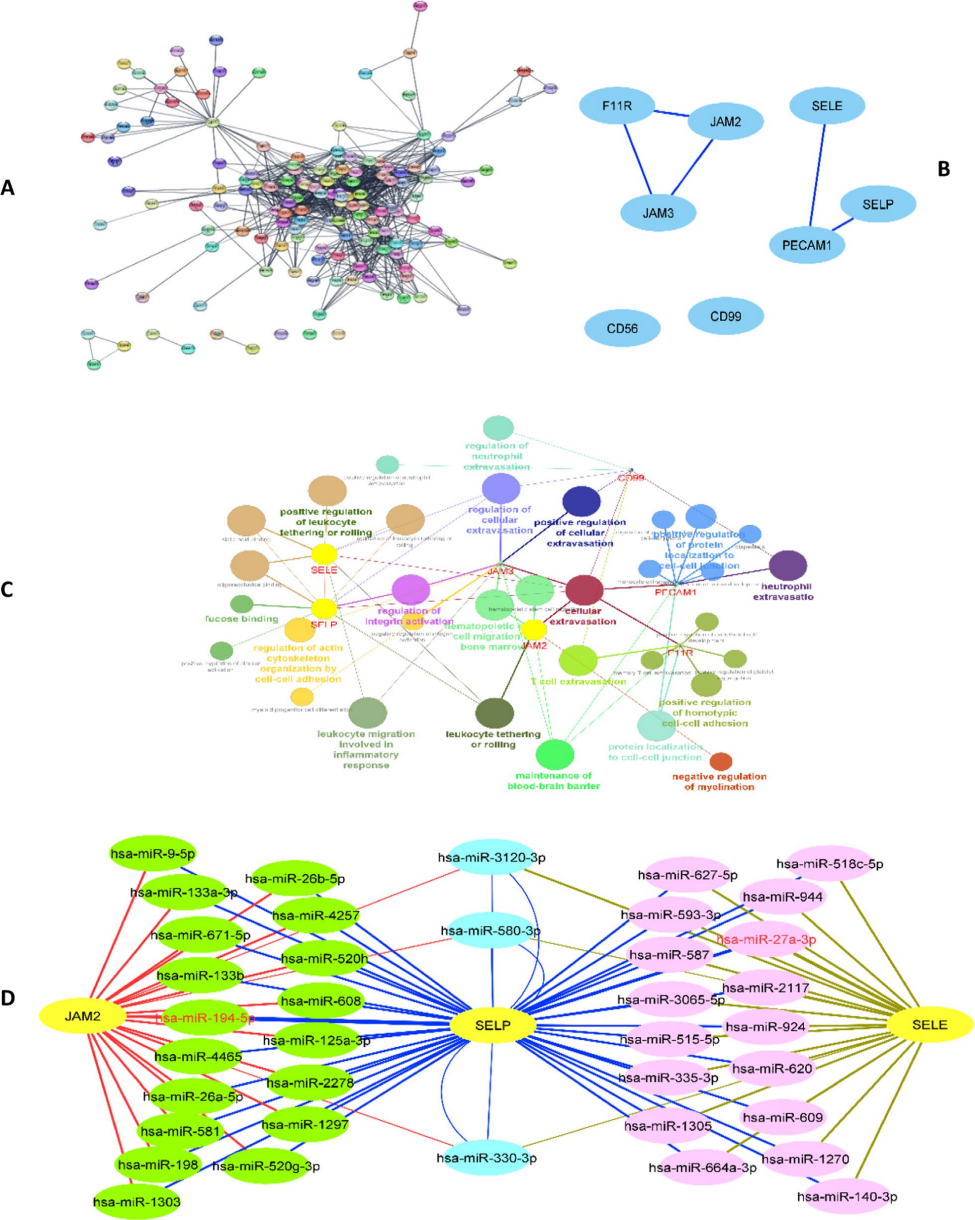
Gene and RNA predictions. **A** Gene network obtained from REACTOME membrane data. **B** The high-score common genes in diapedesis pathway. **C** GO enrichment. **D** Gene-miRNA network

The membrane genes were extracted from REACTOME and were improved on the STRING server (Fig. 1A). The gene data were merged with the diapedesis pathway (KEGG, hsa04670) and were presented as clusters with high-edge nodes using Cytoscape (Fig. 1B). Then, the cluster genes were enriched using GO (molecular function, molecular process) so that three genes (JAM2, SELP, and SELE) were found to be involved with leucocyte tethering on rolling function and process (Fig. 1C). In the other hand, the genes were subjects to search in miRNA databases through miRWalk server. The primary gene-miRNA networks were evaluated and trimmed on the high-frequency reports of the databases so that several miRNAs were predicted to relate to the function of genes. The high-edge miRNAs containing miR-194-5p and miR-27a-3p were selected for the study (Fig. 1D) [23, 24].

#### Statistical analysis

Data were analyzed using Graphpad Prism (Version 8.0.3, San Diego, CA). The significant changes in the study groups were detected by ANOVA followed with Dunn’s post hoc test. P-values < 0.05 were considered as significant levels. 2^−ΔΔCt^ method was used to investigate the gene expression values.

### Results

#### Flow cytometry

**Fig. 2:**
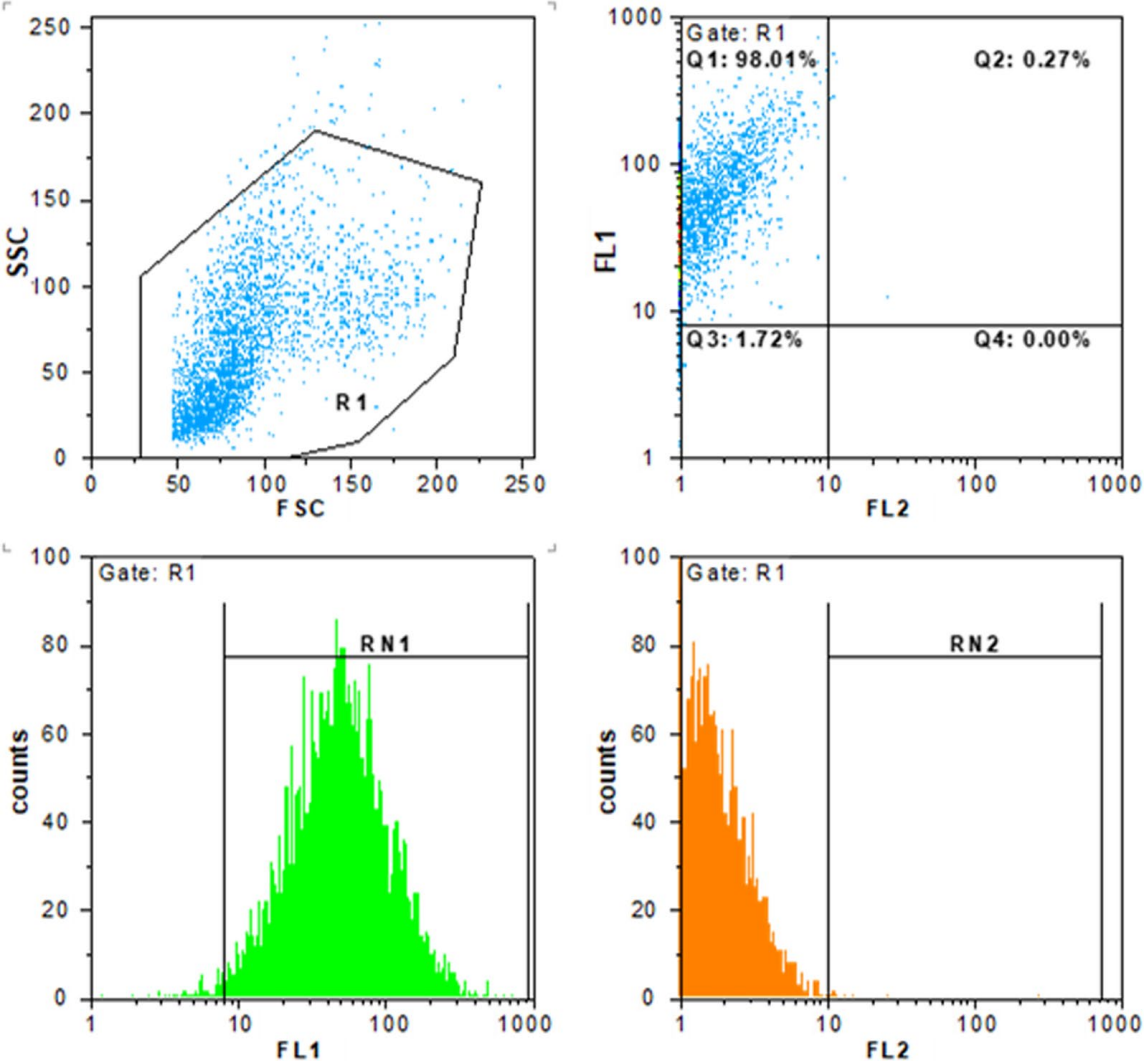
miRNA transfection in human umbilical vein endothelial cells. The PEI particles containing FITC-conjugated miRNA were transfected into cells and the delivery levels were estimated up to 98% using flow cytometry. Scattered light plot of SSC (side scatter) vs FSC (forward scatter). FL1 (fluorescence channel 1) vs. FL2 (orange channel 2) scatter and count plots

The cellular uptake of FITC-labeled PEI/miR complex showed that the miRNA delivery was above 98% in human umbilical vein endothelial cells (Fig. 2).

#### Gene expression

##### miR-194 and miR-27a decreased SELP gene expression level

**Fig. 3:**
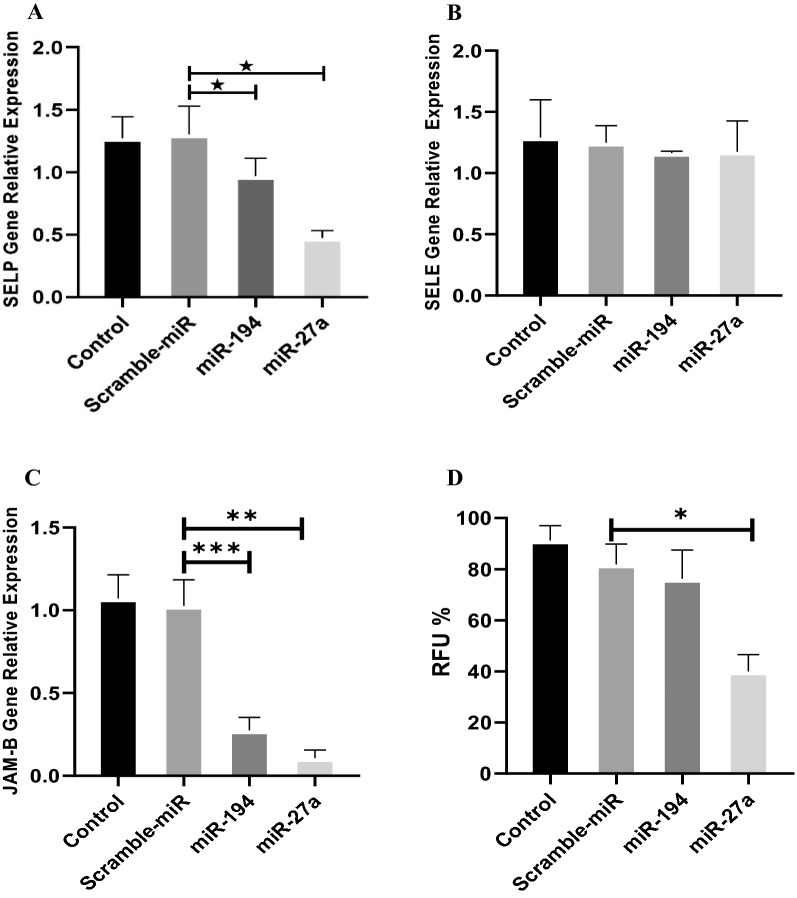
The gene expression levels and monocyte adhesion in HUVECs. The gene expression levels of SELP (**A**), SELE (**B**) and JAM-B (**C**). **D** Monocyte-endothelial adhesion. The molecular adhesion was evaluated in the miRNA-transfected HUVEC cells cocultured with monocytes. Relative Fluorescence Unit (RFU %) as the index of cellular adhesion level. *P-value < 0.05, **P-value < 0.0001, ***P-value = 0.0005

The changes in SELP gene expression levels were significant in the cells treated with miR-194 (P = 0.037). Also, miR-27a decreased significantly the SELP gene expression levels (P = 0.0250) (Fig. 3-A).

##### miR-194 and miR-27a did not change SELE gene expression level

The miR-194 and miR-27a had no significant effects on the SELE gene expression levels (P = 0.9920 and P = 0.9945, respectively) (Fig. 3B).

##### miR-194 and miR-27a decreased JAM-B gene expression level

miR-194 and miR-27a decreased significantly the JAM-B gene expression levels in HUVEC cells (P = 0.0005 and P < 0.0001, respectively) (Fig. 3C).

##### miR-27a inhibited significantly monocyte-endothelial Adhesion

Data on monocyte-endothelial adhesion revealed that miR-27a inhibits significantly (P < 0.0001) monocyte adhesion to endothelial cells. miR-194, however, reduced the monocyte-endothelial adhesion but there was not significant difference as considered to miR-27a (P = 0.5243) (Fig. 3-D).

### Discussion

The activated endothelial cells might overexpress adhesion molecules and enhance leukocyte adhesion following rolling, strong adhesion, crawling, and transmigration processes [7]. Many studies showed that non-coding RNAs target the adhesion molecules and other genes involved in vessel stenosis [20–24]. This study was focused on the effects of miRNA-related genes predicted in the monocyte diapedesis process. Some studies reported that circulating miR-27a decreases in patients with atherosclerosis and suggested to be a biomarker in diagnosis and follow-up of these patients [25]. Moreover, it is suggested that miR-27a shifts the monocyte polarization to macrophage M2 and affects plaque formation [22]. Sun Y et al. showed miR-27a suppresses FADD and prevents from HUVEC apoptosis [26]. Wang Y et al. reported miR-27a inhibits inflammation and cell adhesion in rat kidney epithelial cells by targeting TLR-4 [27]. Romay MC et al. reported miR-27a and miR-21 modulate NF-κB signaling pathway and inhibit the expression of adhesion molecules such as SELE and VCAM-1 in HAEC cells [28]. Wu XY et al. showed that miR-155 targets directly P-65 in NF-κB signaling pathway and inhibits the adhesion of monocytes to HUVECs [29]. miR-27a and b also regulate endothelial adhesion and angiogenesis by suppression of NF‐κB and SEMA6A [30–32]. In agreement with above reports, this study showed that the miR-27a decreases the SELP and JAM-B gene expression levels. This study also showed that miR-27a suppresses significantly the monocyte-endothelial adhesion. However, miR-27a cannot change the E-selectin expression level. It is well known that SELP and JAM-B express by endothelial cells in inflammatory events and bind to monocytes during diapedesis process [16, 18, 19, 33]. Moreover, the results showed that the miR-194 downregulates the JAM-B and SELP gene expression levels in endothelial cells. In agreement with these results, other studies on miR-194 reported that it suppresses the TGF-β/SMAD signaling pathway by targeting THBS1 resulting in the inhibition of inflammation and vasopermeability [34]. Chen R et al. showed that miR-194 inhibits CXCR4 and decreases the expression of IL-1β, IL-6, and TNF-α [35]. In this study, the monocyte-endothelial adhesion was inhibited by miR-194 but not significantly. This phenotype finding might be because more suppression of JAM-B and SELP genes by miR-27a as compared with the miR-194.

Taken together, the prediction results showed that the SELP, JAM-B, and SELE genes might effectively affect the leukocyte diapedesis process by miR-27a and miR-194. The experimental results confirmed the effects of miR-27a and miR-194 on the suppression of the SELP and JAM-B gene expression levels. However, there were no correlations between SELE and miRNAs due to the predicted low-score edges as compared with other edges. Furthermore, the data found a relation between miR-27a and the JAM-B expression levels that were not considered in prediction results.

### Conclusion

The results showed that the miR-27a inhibits effectively the monocyte-endothelial adhesion by suppressing the adhesion molecules in HUVEC cells.

### Limitation

The work is validated on the prediction studies. The results can improve by the focus on the molecular mechanisms between the studied miRNAs and genes and to experience their roles in interventional experiments.

## Data Availability

The data were presented on the request from corresponding Author (Dr. Mohammad Najafi).

## Abbreviations

CAD: Coronary artery diseases
PAD: Peripheral artery diseases
CVD: Cerebrovascular diseases
Ox-LDL: Oxidized-LDL
ROS/RNS: Reactive Oxygen/Nitrogen Species
VSMCs: Vascular smooth muscle cells
ECs: Endothelial cells
SELE: E-selectin
SELP: P-selectin
JAMs: Junctional adhesion molecules
MCP-1: Monocyte chemotactic protein-1
M-CSF: Macrophage colony-stimulating factor
miRNA/miR: MicroRNAs
ECM: Extracellular matrix
GO: Gene ontology

## References

[1] Wolf D, Ley K (2019). Immunity and inflammation in atherosclerosis. Circ Res.

[2] Frostegård J (2013). Immunity, atherosclerosis and cardiovascular disease. BMC Med.

[3] Libby P, Ridker PM, Hansson GK (2009). Inflammation in atherosclerosis. J Am Coll Cardiol.

[4] Malekmohammad K, Sewell RD, Rafieian-Kopaei M (2019). Antioxidants and atherosclerosis: mechanistic aspects. Biomolecules.

[5] Geovanini GR, Libby P (2018). Atherosclerosis and inflammation: overview and updates. Clin Sci.

[6] Tousoulis D (2011). Pathophysiology of atherosclerosis: the role of inflammation. Curr Pharm Des.

[7] Gerhardt T, Ley K (2015). Monocyte trafficking across the vessel wall. Cardiovasc Res.

[8] Čejková S, Králová-Lesná I, Poledne R (2016). Monocyte adhesion to the endothelium is an initial stage of atherosclerosis development. Cor Vasa.

[9] Chamorro-Jorganes A, Araldi E, Suárez Y (2013). MicroRNAs as pharmacological targets in endothelial cell function and dysfunction. Pharmacol Res.

[10] Zampetaki A, Dudek K, Mayr M (2013). Oxidative stress in atherosclerosis: the role of microRNAs in arterial remodeling. Free Radical Biol Med.

[11] Schmitt C (2015). First-in-man study with inclacumab, a human monoclonal antibody against P-selectin. J Cardiovasc Pharmacol.

[12] Wu M-Y (2017). New insights into the role of inflammation in the pathogenesis of atherosclerosis. Int J Mol Sci.

[13] Zhong L, Simard MJ, Huot J (2018). Endothelial microRNAs regulating the NF-κB pathway and cell adhesion molecules during inflammation. FASEB J.

[14] Yao L (1996). Interleukin 4 or oncostatin M induces a prolonged increase in P-selectin mRNA and protein in human endothelial cells. J Exp Med.

[15] Galkina E, Ley K (2007). Vascular adhesion molecules in atherosclerosis. Arterioscler Thromb Vasc Biol.

[16] Weber C, Fraemohs L, Dejana E (2007). The role of junctional adhesion molecules in vascular inflammation. Nat Rev Immunol.

[17] Kummer D, Ebnet K (2018). Junctional adhesion molecules (JAMs): the JAM-integrin connection. Cells.

[18] Luissint A-C, Nusrat A, Parkos CA. JAM-related proteins in mucosal homeostasis and inflammation. In: seminars in immunopathology. 2014; Springer. 10.1007/s00281-014-0421-0 PMC4084508 24667924

[19] Lamagna C (2005). Dual interaction of JAM-C with JAM-B and αMβ2 integrin: function in junctional complexes and leukocyte adhesion. Mol Biol Cell.

[20] Poursaleh A, Beigee FS, Esfandiari G, Najafi M (2021). Adhesion of monocytes and endothelial cells isolated from the human aorta suppresses by miRNA-PEI particles. BMC Cardiovasc Disord.

[21] Ghasempour G, Shaikhnia F, Soleimani AA, Rahimi B, Najafi M (2021). Correlations between vitronectin, miR-520, and miR-34 in patients with stenosis of coronary arteries. Mol Biol Rep.

[22] Feinberg MW, Moore KJ (2016). MicroRNA regulation of atherosclerosis. Circ Res.

[23] Ghasempour G, Mohammadi A, Zamani-Garmsiri F, Najafi M (2021). miRNAs through β-ARR2/p-ERK1/2 pathway regulate the VSMC proliferation and migration. Life Sci.

[24] Ghasempour G, Mahabadi VP, Shabani M, Mohammadi A, Zamani-Garmsiri F, Amirfarhangi A, Karimi M, Najafi M (2021). miR-181b and miR-204 suppress the VSMC proliferation and migration by downregulation of HCK. Microvasc Res.

[25] Telkoparan-Akillilar P, Cevik D (2021). Identification of miR-17, miR-21, miR-27a, miR-106b and miR-222 as endoplasmic reticulum stress-related potential biomarkers in circulation of patients with atherosclerosis. Mol Biol Rep.

[26] Sun Y (2019). miR-27a regulates vascular remodeling by targeting endothelial cells' apoptosis and interaction with vascular smooth muscle cells in aortic dissection. Theranostics.

[27] Wang Y, Wang D, Jin Z (2019). miR-27a suppresses TLR4-induced renal ischemia-reperfusion injury. Mol Med Rep.

[28] Romay MC (2015). Regulation of NF-κB signaling by oxidized glycerophospholipid and IL-1β induced miRs-21-3p and-27a-5p in human aortic endothelial cells. J Lipid Res.

[29] Wu XY (2014). Regulation of microRNA-155 in endothelial inflammation by targeting nuclear factor (NF)-κB P65. J Cell Biochem.

[30] Madrigal-Matute J (2013). MicroRNAs and atherosclerosis. Curr Atherosclerosis Rep.

[31] Urbich C (2012). MicroRNA-27a/b controls endothelial cell repulsion and angiogenesis by targeting semaphorin 6A. Blood J Am Soc Hematol.

[32] Li J, Li K, Chen X (2019). Inflammation-regulatory microRNAs: valuable targets for intracranial atherosclerosis. J Neurosci Res.

[33] Woollard KJ (2008). Pathophysiological levels of soluble P-selectin mediate adhesion of leukocytes to the endothelium through Mac-1 activation. Circ Res.

[34] Qu S, Yang L, Liu Z (2020). MicroRNA-194 reduces inflammatory response and human dermal microvascular endothelial cells permeability through suppression of TGF-β/SMAD pathway by inhibiting THBS1 in chronic idiopathic urticaria. J Cell Biochem.

[35] Chen R (2020). Suppressed nuclear factor-kappa B alleviates lipopolysaccharide-induced acute lung injury through downregulation of CXCR4 mediated by microRNA-194. Respir Res.

